# The impacts of putting the Zaporizhzhia Nuclear Power Plant in the line of fire

**DOI:** 10.1002/puh2.26

**Published:** 2022-10-17

**Authors:** Christos Tsagkaris, Lolita Matiashova, Anna Isayeva

**Affiliations:** ^1^ Public Health and Policy Working Group European Student Think Tank Amsterdam The Netherlands; ^2^ LT Malaya Therapy National Institute National Academy of Medical Sciences of Ukraine Kharkiv Ukraine

**Keywords:** food security, nuclear energy, nuclear power plant, radiation, Russia, Ukraine, war

## Abstract

The armed forces of the Russian Federation captured the Zaporizhzhia Nuclear Power Station after their invasion to Ukraine in February 2022. In early August 2022, reports about shelling of the station ignited fear of a nuclear accident. Simultaneously, the disruption in the generation of electricity from nuclear energy has fueled a notable increase in the price of oil. The compounding effect of these developments on global health includes acute and chronic radiation exposure, food insecurity due to the potential contamination of the environment and the rise in the cost of essential commodities, and limited access to healthcare. International and national governance bodies need to take action in order to mitigate the effects of this incident on health and prevent similar events in the future.

On February 24, 2022, the armed forces of the Russian Federation invaded Ukraine and seized the southern part of the Zaporizhzia district, including the Zaporizhzhia Nuclear Power Station. The Power Station is located near the city of Enerhodar on the southern bank of the Dnipro River in southeastern Ukraine. Consisting of six pressurized light water nuclear reactors, it is the biggest power plant in Europe and generates approximately 20% of the electrical power used in Ukraine [1]. The risk of damage to the reactor awoke memories of the nuclear catastrophe in Chernobyl in 1986 and alarmed the international community.

In early March 2022, shelling damaged buildings in the periphery of the station. The reactors sustained no damage and there was no increase in radiation levels in the area. Since then, the station has been controlled by the Russian Agency for Atomic Energy. The Russian side allegedly installed rocket launchers in the station in July 2022 aiming to avoid retaliation by using the critical infrastructure as a shield. However, in early August 2022, a barrage of shelling was reported in the area. This caused damage to the fire station of the plant and lead to the disconnection of several reactors from the power grid. As of August 3, 2022, the International Atomic Energy, the Government of Ukraine, and Ministers from the G7 group of nations have urged for an inspection of the site by international experts and cessation of military activities in its surroundings [2].

The war in Ukraine has already affected individual and population health locally and globally in many manners. These range from combat injuries and wound infections to food insecurity [3]. The development of the situation around the nuclear power station can have a dire and immediate impact on multiple sectors of global health. A potential nuclear accident would have acute and chronic health effects. Doses equivalent or greater than 20 Gray (Gy) of ionizing radiation can cause severe nausea, vomiting, skin burns, immune deficiency, and loss of conscience within minutes after exposure to radiation (acute radiation syndrome) [4]. In the long‐term people, particularly children and youth, exposed to radiation are at higher risk of developing cancer, cardiovascular and musculoskeletal diseases [5]. In addition to the station's personnel and the residents in the neighboring areas, depending on the environmental conditions, radiation leak from the plant can spread within and outside Ukraine as shown in Figure 1.

**FIGURE 1:**
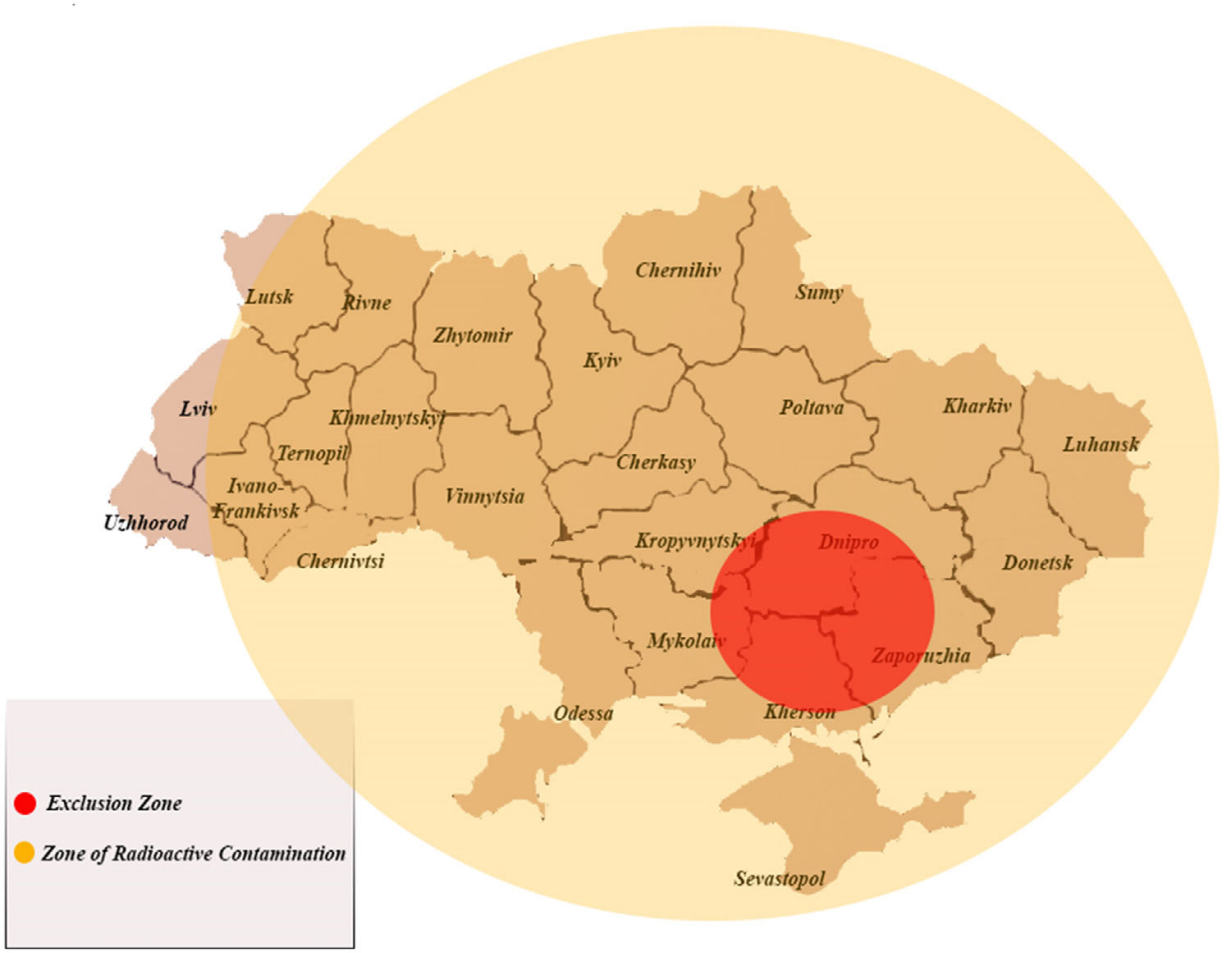
The geographic spread of radioactive debris in an event of a nuclear accident in the Zaporhizhzhia Nuclear Power Station

Debris mixed with radionuclides can be spread through the soil, the air, and the water. Soil contamination in Ukraine can degrade grain and agricultural produce that are distributed globally. Consuming or dumping these products comes with a higher risk of radiation toxicity or starvation in many low‐ and middle‐income countries (LMICs) such as Somalia, Benin, Egypt, Sudan, and Laos, which import most of their wheat supply from Ukraine [6]. The proximity of the power plant to the Dnipro River and the Black Sea poses a threat to neighboring countries such as Georgia, Romania, Bulgaria, and Turkey. Radioactive waste from Chernobyl is still detected in the Black Sea [7]. Further pollution could increase the risk of excess cancer and cardiovascular diseases in the region. It can have grave effects on the fishing industry and contribute to a global food crisis. Studies investigating the nuclear accident in Fukoshima, Japan, showed that levels of radiation in the ocean and in seafood did not decline for at least one year after the event [8]. The risk of radiation exposure along the coasts of the Black Sea can cause a notable decrease in tourism which is a major source of income for these communities [9]. The health, radiation, environmental, food, and financial impacts of a nuclear accident at the Zaporizhzhia power station on Ukraine; its neighboring countries; and the global community are summarized in Figure 2.

**FIGURE 2:**
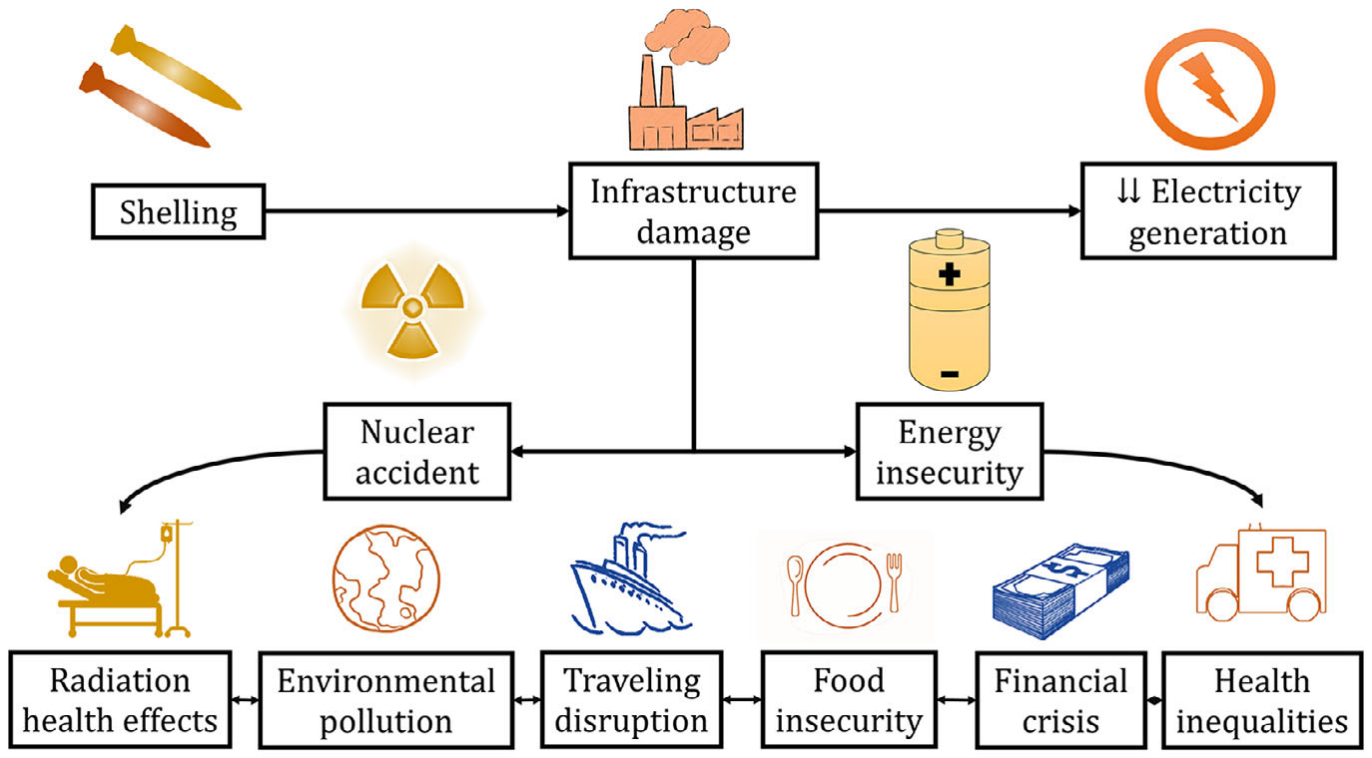
An overview of the impacts of nuclear accidents on energy security and global health

Although a nuclear accident remains an undesired misfortune, the incidents around the Zaporizhzhia power station have already affected the global energy market. The disruption of the station's function has depleted Ukraine of approximately ⅕ of its electricity supply. Filling this deficit with other energy sources has reportedly triggered a stark increase in oil prices across Asian Markets [10]. In a world where oil constitutes both a financial asset and a barometer of global economic activity, this development has a significant contribution in causing an increase in prices of essential goods [11]. To date, the war has already fueled inflation to an extent that people in LMICs face food shortages, while people in high‐income countries experience an unprecedented rise in the value of food, housing, and insurance. It is also known that a shift in oil prices leads to increases in healthcare expenditure due to an increase in the costs of healthcare‐associated supply chains, energy for healthcare facilities, petrol‐based healthcare equipment (implants, pills, personal protective equipment), and the transportation cost of patients and healthcare personnel [12]. Such barriers to access healthcare combined with the adverse health effects of food insecurity and the COVID‐19 morbidity can exacerbate healthcare inequalities across the globe.

While the situation on the ground remains unpredictable, experts suggest that the risk of a nuclear accident is low. A number of structural safety valves of the Zaporizhzhia nuclear power station can prevent the leakage of radiation in case shelling befalls the nuclear reactors. On the other hand, the adaptation of the energy market to the situation of the global economy, and the consequences on health and healthcare should not be underestimated. International and national governance bodies need to urgently extend their help towards preserving global health and wellbeing. There is a need for timely risk assessment by experts, improving access of the public to true and credible information, radiation accidents contingency plans, and emergency funding for equal access to essential goods and healthcare in the affected region are pivotal. In the long term, more efforts towards alternative sources of energy (sustainable energy) and food are needed.

## AUTHOR CONTRIBUTIONS

*Conceptualization*: Christos Tsagkaris. *Writing*: Christos Tsagkaris and Lolita Matiashova. *Revision and response to peer review comments*: Christos Tsagkaris. *Literature search*: Lolita Matiashova. *Critical revision*: Anna Isayeva. *Supervision*: Anna Isayeva.

## CONFLICT OF INTEREST

The authors declares that there is no conflict of interest .

## ETHICS APPROVAL

This letter does not involve patients, animals or sensitive data. It is based on publicly available peer – reviewed studies, gray literature and news reports. Ethical approval was not deemed necessary.

## Data Availability

No data were generated.
